# *Bletilla striata* Oligosaccharides Improve Ulcerative Colitis by Regulating Gut Microbiota and Intestinal Metabolites in Dextran Sulfate Sodium-Induced Mice

**DOI:** 10.3389/fphar.2022.867525

**Published:** 2022-04-25

**Authors:** Tianxiang Zhu, Baifei Hu, Cheng Ye, Haiming Hu, Mingzhu Yin, Zhigang Zhang, Shuiqing Li, Yanju Liu, Hongtao Liu

**Affiliations:** ^1^ College of Basic Medicine, Hubei University of Chinese Medicine, Wuhan, China; ^2^ Wuhan Customs Technology Center, Wuhan, China

**Keywords:** *Bletilla striata* oligosaccharides, ulcerative colitis, gut microbiota, intestinal metabolites, intestinal barrier

## Abstract

This study aimed to elucidate the mechanism of *Bletilla striata* oligosaccharides (BO) in the treatment of ulcerative colitis (UC). A UC mouse model was induced by 3% Dextran sodium sulfate (DSS), and BO (200 mg/kg/d) were administered for intervention. The results show that BO effectively inhibited the release of intestinal inflammatory cytokines such as IL-6, TNF-α, and IL-1β. Also, BO profoundly elevated the secretion of mucins and the expression of tight junction (TJ) proteins to attenuate dysfunction of the intestinal barrier. The 16S rDNA sequencing and liquid chromatography/gas chromatography-mass spectrometer (LC/GC-MS) analysis of mouse feces revealed that BO regulated the disturbance of gut microbiota and intestinal metabolites. By using the *in vitro* fermentation broth of BO and gut microbiota-depleted mice treated with antibiotics, we confirmed the protection of BO against UC. In conclusion, BO played a role in improving UC by modulating gut microbial composition and intestinal metabolites, which provided new therapeutic strategies for UC treatment.

## Introduction

Ulcerative colitis (UC) is an idiopathic and chronic inflammatory bowel disease (IBD), and the main symptoms are episodes of abdominal pain and bloody diarrhea (Feuerstein et al., 2019). Patients with UC may experience intestinal perforation in severe cases. UC is characterized by a long course of the disease, difficulty in curing, and various complications, which seriously affect the life quality of patients. According to statistics, the incidence of UC increased year by year. In China, patients with IBD will reach 1.5 million by 2025 (Kaplan, 2015). In recent years, the primary clinical drugs for UC treatment included 5-aminosalicylic acid, corticosteroids, immunosuppressants, and monoclonal antibodies, but these drugs may have clinical limitations or even severe side effects (Eisenstein, 2018). This makes it more urgent to explore new therapeutic candidates and methods.

The pathogenesis of UC is related to impaired intestinal barrier, abnormal immune response, dysbiosis of gut microbiota, genetic mutation, and environmental changes (Porter et al., 2020). Among them, intestinal flora disturbance is a crucial contributor to the occurrence of UC (Sheehan et al., 2015). Gut microbiota exerts an essential regulatory role in maintaining the physiological activities of hosts under normal circumstances. At the same time, the imbalance between beneficial and harmful bacteria will destroy the intestinal barrier and thus induce or aggravate the development of UC (Pei et al., 2019). For example, the abundance of adherent-invasive *Escherichia coli*, *Clostridium,* and *Fusobacterium* increased. In contrast, the contents of *Faecalibacterium prausnitzii*, *Bifidobacterium*, and *Roseburia* decreased significantly in UC patients’ intestines (Zhang et al., 2017). Metabolites of gut microbiota, like bile acids (BAs), short-chain fatty acids (SCFAs), and tryptophan catabolites, are also involved in the pathogenesis of UC (Louis et al., 2014). During UC progression, BA synthesis, transport, and excretion in the liver are aberrant, leading to the accumulation of BAs in the intestine and causing intestinal inflammation (Negroni et al., 2020). Evidence demonstrated that deficiency of secondary BAs, which are generated by hydrolysis of intestinal flora, may disrupt the intestinal mucosal integrity and exacerbate the severity of colitis (Sinha et al., 2020). SCFAs, including acetic acid, propionic acid, and butyric acid, are produced by gut microbial metabolism of indigestible fiber-rich diets (Sun et al., 2017). It was reported that butyric acid improved the intestinal barrier dysfunction and offered adequate treatment of DSS-induced colitis by regulating autophagy through Hypoxia-inducible factor-1α (HIF-1α) (Zhou et al., 2020). Tryptophan, an essential amino acid in humans, is metabolized by gut microbiota to produce indole-3-ethanol, indole-3-pyruvate, and indole-3-aldehyde. All of these catabolites initiate protective functions on the intestinal barrier. Thus, it might be a new strategy to improve UC by modulating gut microbiota homeostasis.

Natural products such as polyphenols, alkaloids, and polysaccharides have been certificated to have favorable anti-UC activity (Liu et al., 2018; Ji et al., 2020). For thousands of years, the traditional Chinese medicine *Bletilla striata* (Thunb.) Rchb. f. [Orchidaceae] has been used in China mainly to treat traumatic bleeding and digestive system disorders (Xu et al., 2019). *B. striata* contains a variety of natural chemical constituents such as polysaccharides, glycosides, phenanthrenes, quinones, and bibenzyls (Xu et al., 2019). Among them, the polysaccharides have the highest content in *B. striata* tuber (Zhang et al., 2019). Luo et al. (2018) discovered that *B. striata* polysaccharide (BP) might be a novel protective agent of the intestinal epithelial barrier. However, due to the complex structure and high viscosity of BP, the in-depth research of BP activity has been limited.

Oligosaccharides are a new type of available glycogen with high solubilities and biological activities. Compared to npolysaccharides, oligosaccharides can be easily decomposed and utilized by multiple intestinal florae, thus influencing the enteric homeostasis to benefit human health (Goh & Klaenhammer, 2015). In this study, we proposed to degrade BP into oligosaccharides effectively and investigated its molecular mechanism for improving UC through the metabolic regulation of gut microbiota.

## Materials and Methods

### Reagents and Antibodies

*B. striata* oligosaccharides (BO) were extracted from *B. striata* (Supplementary Methods; Supplementary Figures S1, S2). The preparation methods were carried out according to the previous report, and the molecular weight of BO was 720–1080 Da (Chen et al., 2019; Hu et al., 2020). *Bletilla striata* (Thunb.) Rchb.f. [Orchidaceae] was purchased from Hubei Zexi Chinese Medicine Technology Co., Ltd. (Qichun, Hubei, China) and authenticated by Xiongjie Sun in Hubei University of Chinese Medicine. Dextran sulfate sodium (DSS, MW 36000–50000) was purchased from MP Biomedicals (Santa Ana, CA, United States). Mouse NGAL (Neutrophil Gelatinase Associated Lipocalin) ELISA kit was bought from Elabscience Biotechnology Co., Ltd. (Wuhan, China). TRIzol Reagent and Biotin-avidin IHC kits were obtained from summer Biotechnology Co., Ltd. (Beijing, China). FastHS SYBR QPCR mixture and AMeasy 1st Strand cDNA synthesis kit were bought from AllMEEK (Beijing, China). Tryptone and yeast extract (YE) were purchased from Amresco (Washington, DC, United States). L-Cysteine, ascorbic acid, ampicillin, vancomycin, neomycin sulfate, metronidazole, and SCFAs (Acetic acid, propanoic acid, butyric acid, and valeric acid) were obtained from Aladdin (Shanghai, China). Tryptamine, 5-hydroxytryptamine (5-HT), indole, Cholic acid (CA), Chenodeoxycholic acid (CDCA) deoxycholic acid (DCA), taurocholic acid (TCA), ursodeoxycholic acid (UDCA), taurochenodeoxycholic acid (TCDCA), cholic acid-2,2,3,4,4-d5 (d_5_-CA) and sodium taurocholate-2,2,4,4-d4 (d_4_-TCA) were purchased from Sigma Aldrich (St. Louis, MO, United States). Tauro-β-murocholicacid (T-β-MCA) was bought from TRC (Toronto, ON, Canada).

Antibodies for extracellular regulated kinase 1 and 2 (ERK1/2), p-ERK, zonula occludens-1 (ZO-1), and Claudin-1 were bought from Santa Cruz Biotechnology (Santa Cruz, CA, United States). Antibodies for NOD-like receptor pyrin domain containing 3 (NLRP3), c-Jun N-terminal kinase (JNK), and p-JNK were purchased from Cell Signaling Technology (Danvers, MA, United States).

### Animal Experiment

Six-week-old Specific pathogen free (SPF) male BALB/c mice (22 ± 2 g) were purchased from Hubei Provincial Center for Disease Control and Prevention (Wuhan, China). All mice were housed under temperature-controlled conditions (12 h light/dark cycle, 22 ± 1°C) with free access to sterile water and standard food. After the acclimation, mice were randomly divided into four groups: 1) Ctrl group, treated with distilled water; 2) DSS group, received 3% DSS (in drinking water) from day 15 to 21; 3) BO group, in which mice were given BO (200 mg/kg/d) by gavage for 21 days; 4) DSS + BO group, in which mice were given BO by gavage from day 1 to 21 and received 3% DSS from day 15 to 21. The dose of BO was based on a study of flaxseed oligosaccharides in colitis (Xu et al., 2020). During the experiment, the weight loss, stool consistency, and gross bleeding were recorded daily for the assessment of disease activity index (DAI) score (Supplementary Table S1). After the treatment, all mice were euthanized, and the major tissues were collected. All samples were stored at −80°C for further analysis. In addition, colons were removed to measure the length, and part of the distal colon was fixed with 4% paraformaldehyde. The animal experiment was performed according to the requirement of the Animal Ethical Experimentation Committee of the Hubei University of Chinese Medicine and the National Act on Use of Experimental Animals (China).

### Fermentation Experiment

The culture medium was prepared based on a simulated intestinal environment medium, the components of which were provided in Supplementary Table S2. After the pH was adjusted to 7.5–7.6, the intestinal flora culture medium was autoclaved at 121°C for 20 min. Filter-sterilized 25% ascorbic acid was added to the medium after high temperature and pressure sterilization. Fresh fecal samples from normal BALB/c mice were evenly dispersed in 1% sterile PBS to obtain 20% (w/v) fecal suspension, followed by centrifugation at 3000 rpm for 5 min. Next, 6 ml of the collected supernatant was added to 54 ml of intestinal flora culture medium (containing 1.8 g BO) with a final ratio of 1:9 (v/v). Then, the fermentation broth was placed in an anaerobic culture tank (including 10% H_2_, 10% CO_2_, and 80% N_2_) and incubated at 37°C for 24 h. After the anaerobic fermentation, the broth was centrifuged at 12,000 rpm for 10 min, and the supernatant was collected and filtered with a 0.22 μm microporous membrane for the animal experiment.

### Antibiotic Treatment

Six-week-old male BALB/c mice were randomly divided into five groups: Ctrl group, DSS group, DSS + Antibiotic mixtures (Abx) group, DSS + Abx + BO group and DSS + Abx + BO fermentation broth (BO FB) group. Abx included ampicillin (1 mg/ml), vancomycin (0.5 mg/ml), neomycin sulfate (1 mg/ml), and metronidazole (0.5 mg/ml). The administration protocol was detailed in Figure 5A, and the administration route and dose were the same as above. The body weight, stool consistency, and rectal bleeding were recorded daily for assessment of DAI score.

Before the BO treatment, fecal samples were collected to extract and quantify bacterial genomic DNA to confirm that the gut microbiota was depleted in mice. The experimental details were provided in Supplementary Methods and Supplementary Table S3. At the end of the animal experiment, the mice were sacrificed, and major tissues were collected as above.

### RNA Extraction and Quantitative Real-Time PCR

Total RNA was isolated from colon tissues using Trizol reagents (summer Bio, Beijing, China). cDNA was generated using an AMeasy 1st Strand cDNA synthesis kit (AllMEEK, Beijing, China). Next, RT-qPCR was performed using a 2 × FastHS SYBR QPCR mixture (AllMEEK, Beijing, China) on a CFX Connect Real-time system (Bio-Rad, Hercules, CA, United States). The amplification protocol was as follows: initial denaturation step at 95°C (15 min), 40 cycles at 95°C (8 s), 60°C (30 s) for amplification. All primer sequences are shown in Supplementary Table S4. Glyceraldehyde-3-phosphate dehydrogenase (GAPDH) was used as an internal reference to normalize the expressions of target genes. The 2^−ΔΔCT^ method was used to calculate the relative mRNA expression.

### Western Blotting

Colon tissues were homogenized using RIPA buffer (Cell Signaling Technology Inc., MA, United States) supplemented with a protease inhibitor cocktail (Merck, Darmstadt, Germany). Protein concentration was measured with a BCA protein assay kit (Thermo Fisher Scientific, Waltham, MA, United States). Protein samples were separated by SDS-PAGE and transferred onto PVDF membranes. The membranes were blocked with 5% skim milk for 1 h and incubated with primary antibodies at 4°C overnight, including NLRP3 (1:1,000, Cat #15101), p-ERK (1:200, Cat #sc-9383), ERK (1:200, Cat #sc-514302), p-JNK (1:1,000, Cat #9251), JNK (1:1,000, Cat #9252), and β-Actin (1:500, Cat #sc-81178). Then, the membranes were washed and interacted with horseradish peroxidase-conjugated secondary antibody (1:5,000) at room temperature for 1.5 h. Finally, the target protein bands were detected by enhanced chemiluminescence (ECL) (Sigma Aldrich, MO, United States). The densitometry analysis was performed using Image J2x software (National Institute of Health, United States).

### Histological Analysis

Colon tissues were fixed with 4% paraformaldehyde, dehydrated, embedded in paraffin, and cut into sections of 5 μm. Hematoxylin and eosin (H&E) staining kit (Beyotime Institute of Biotechnology, Shanghai, China) was used for slide staining. The production of intestinal mucins was assayed by staining with an Alcian Blue staining kit (Vectorlabs, Beijing, China) and fluorescein isothiocyanate conjugated-wheat germ agglutinin (WGA-FITC) immunofluorescence (Sigma Aldrich, MO, United States). The immunohistochemistry of colon tissues was analyzed using anti-ZO-1 (1:50, Cat #sc-33725) and anti-Claudin-1 (1:50, Cat #sc166338) antibodies. Images of colon structures were obtained by a Leica DMIL 4000B light microscope with a Leica DFC450C digital camera (Wetzlar, Germany).

### Analysis of Intestinal Metabolites in Feces

For the quantification of fecal BAs, 50 mg of stool samples were dissolved in 1 ml water-methanol-formic acid solution (25:74:1, V/V/V) containing d5-CA and d4-TCA as internal standards. The mixture was homogenized and centrifuged at 12,000 ×g for 15 min at 4°C. After filtration with 0.22 μm microporous membranes, all samples were analyzed by a liquid chromatography-mass spectrometer (LC-MS). The LC-MS parameters referred to previous studies (Zheng et al., 2021). For the analysis of SCFAs and tryptophan metabolites, 50 mg of stool sample was dissolved in 1 ml 50% (V/V) methanol aqueous solution (containing 0.2% HCl). After the treatment as above, all samples were analyzed by LC-MS or gas chromatography-mass spectrometer (GC-MS). The detailed LC/GC-MS parameters were provided in Supplementary Methods.

### 16S rDNA Sequencing of Gut Microbiota

Fast DNA™ SPIN Kit (MP Biomedicals, CA, United States) was used to extract fecal bacterial genomic DNA. Barcoded conventional primers (forward 338 F, 5′-ACT​CCT​ACG​GGA​GGC​AGC​AG-3′; reverse 806 R, 5′-GACTACHVGGGTWTCTAAT-3′) were applied to amplify V3–V4 hypervariable regions of bacterial 16S rDNA gene by RT-qPCR. The reaction process was as follows: an initiation at 95°C for 5 min, 20 cycles at 95°C for 30 s, 55°C for 30 s and 72°C for 30 s. Finally, the reaction was extended 10 min at 72°C. The purified amplicons were pooled in paired-end sequencing (2 × 300) on an Illumina MiSeq platform (Illumina, San Diego, CA, United States) by Beijing Allwegene Tech (Beijing, China). The data of high-quality sequence was analyzed using QIIME package (Quantitative Insights into Microbial Ecology, United States) (Version 1.8, http://qiime.org). The main criteria for selecting sequences were nucleotide ≥110, sequence overlaps ≥10 bp, an average quality score higher than 20 in a sliding window of 50 bp, an exact match to primers, and clear features. UCLUST (Version 1.2.22, http://www.Drive5.com/uclust/downloads1-2-22q.html) divided the unique sequence into an operational taxonomic unit (OTU) when the similarity is more significant than 97%. Chimeric sequences were screened and removed using Usearch (Version 8 January 1861, http://www.drive5.com/usearch). The taxonomy of each 16S rDNA gene sequence was identified by UCLUST against the Silva (Release 128 http://www.arb-silva.de) and Greengene 16S rRNA database (Release 13.5, http://greengenes.secondgenome.com/), with a minimum threshold of 90% confidence for identification. At last, an OTU table was generated for gut microbiota composition and abundance analysis. Functional prediction of bacterial communities was finished by a bioinformatic tool (PICRUSt, Phylogenetic Investigation of Communities by Reconstruction of Unobserved States). Spearman’s rank correlation coefficient analyzed the Correlation between intestinal flora and different indicators.

### Statistical Analysis

Results were presented as mean ± SD. Statistical differences were evaluated by one-way analysis of variance (ANOVA) followed by Tukey-Kramer test among groups. It was significant for all statistical analyses that the probability value was less than 0.05. Data were calculated using Prism version 8.0 GraphPad Software.

## Results

### Improvement of *Bletilla striata* Oligosaccharides on Physiological Indices in UC Mice

**FIGURE 1:**
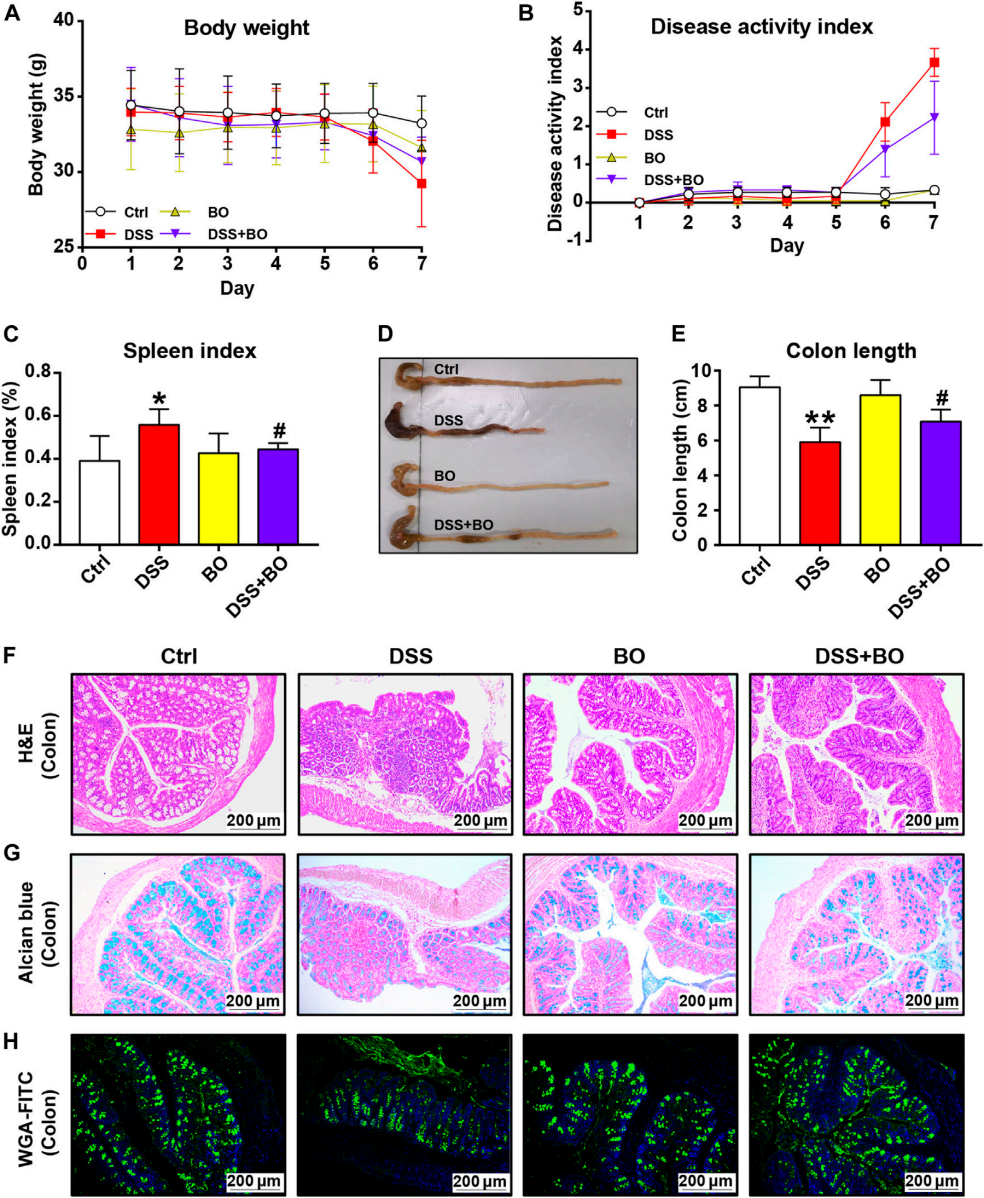
BO ameliorated pathological indices of UC mice. **(A)** Changes in body weight during disease progression. **(B)** DAI scores. **(C)** Spleen index. **(D)** Photographs of the colon. **(E)** Colon length. **(F)** Hematoxylin & eosin (H&E) staining for morphological structure of colon tissues. Scale bar = 200 μm. **(G,H)** Glycoprotein change of mucins in the colon stained with Alcian blue **(G)** and wheat germ agglutinin labeled with FITC. **(H)** Scale bar = 200 μm. Data were shown as mean ± SD (*n* = 6). *p < 0.05, ^**^ *p* < 0.01 vs. Ctrl group; ^#^ *p* < 0.05, ^##^ *p* < 0.01 vs. DSS group.

In the present study, DSS was used to induce UC in mice. From the fourth day of DSS administration, the mice exhibited evident body weight loss, and the weight loss was slowed down by BO treatment (Figure 1A). DAI scores were performed to determine the severity of UC based on body weight loss, stool consistency, and gross bleeding (Murthy et al., 1993). As shown in Figure 1B, the DAI scores in UC mice dropped after BO administration. Also, BO were found to inhibit the increase of spleen index in UC mice (*p* < 0.05, vs. DSS group) (Figure 1C). Meanwhile, a pronounced decrease in the length of the colon was observed in UC mice, and this was expectedly rescued by BO (*p* < 0.05, vs. DSS group) (Figures 1D,E). Furthermore, H&E staining of colon tissues demonstrated that the muscular layer and crypt of colon tissues were severely disrupted, accompanied by an absence of goblet cells and massive infiltration of inflammatory cells in UC mice, while these damages were significantly restored by BO treatment (Figure 1F). The glycoprotein change in mucins was detected by Alcian blue and WGA-FITC staining (Figures 1G,H). It was shown that BO reversed the reduction of glycoprotein mucins in the colon of UC mice. Lipocalin-2 (Lcn2) is an emerging clinically significant biomarker for IBD. Therefore, the serum Lcn2 in UC mice were determined using an ELISA kit. The results demonstrated that BO reduced the content of serum Lcn2 in UC mice (Supplementary Figure S3).

### Inhibitory Effect of *Bletilla striata* Oligosaccharides on Intestinal Inflammation and Intestinal Barrier Damage in UC Mice

**FIGURE 2:**
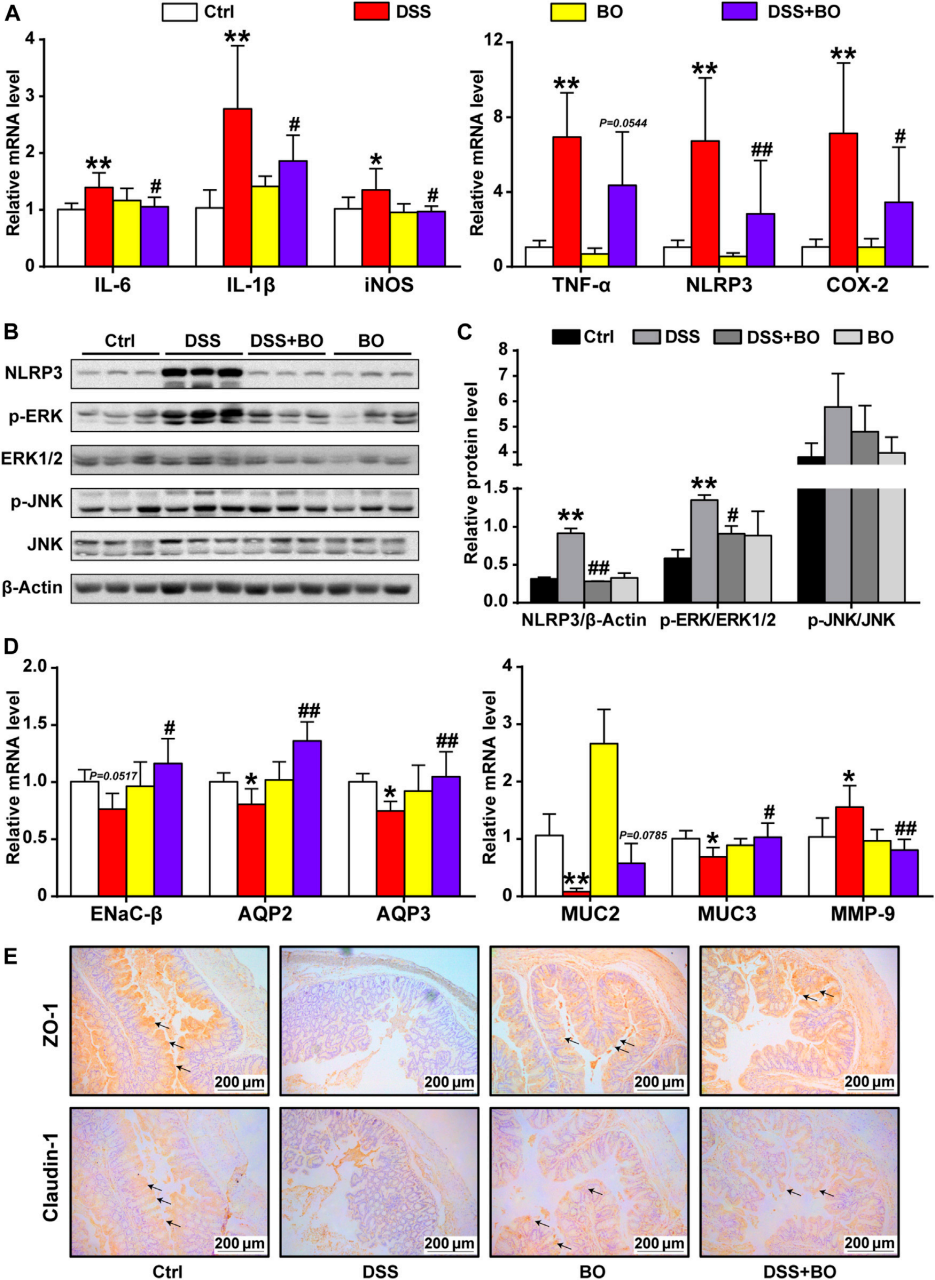
BO inhibited inflammatory responses and gut barrier damage in the colon of UC mice. **(A)** mRNA levels of inflammatory factors by RT-qPCR, including IL-6*,* IL-1β, iNOS, TNF-α, NLRP3, and COX-2. **(B)** Protein expressions of NLRP3, p-ERK, ERK1/2, p-JNK, and JNK in colon tissues by WB. **(C)** Relative intensities of NLRP3, p-ERK, and p-JNK at protein levels. **(D)** Expressions of regulators related to aqueous metabolism, intestinal integrity, and gut barrier at mRNA levels, including ENaC-β, AQP2, AQP3, MUC2, MUC3, and MMP-9. **(E)** Immunohistochemical analysis of ZO-1 and Claudin-1 in colon tissues. Scale bar = 200 μm. Data were shown as mean ± SD (*n* = 6). *p < 0.05, **p < 0.01 *vs.* Ctrl group; ^#^ *p* < 0.05, ^##^ *p* < 0.01 vs. DSS group.

Next, RT-qPCR, WB, and immunohistochemistry were adopted to assess the protective effect of BO on colon tissues. Compared to the DSS group, BO inhibited the overexpression of interleukin-6 (IL-6), interleukin-1β (IL-1β), inducible nitric oxide synthase (iNOS), tumor necrosis factor-α (TNF-α), NOD-like receptor pyrin domain containing 3 (NLRP3), and cyclooxygenase-2 (COX-2) in UC mice (*p* < 0.05 or 0.01, vs. DSS group) (Figure 2A). At the protein level, BO not only reduced the expression of NLRP3 but also blocked the activation of ERK and JNK in the colon of UC mice (*p* < 0.05 or 0.01, vs. DSS group) (Figures 2B,C).

We also assessed the effect of BO treatment on abnormal intestinal fluid metabolism (an indicator of severe diarrhea) in UC mice. As indicated in Figure 2D, the expression of epithelial sodium channel-β (ENaC-β), aquaporin 2 (AQP2), and aquaporin 3 (AQP3) was curbed in the DSS group (*p* < 0.01, vs. Ctrl group). In contrast, BO treatment significantly promoted the expressions of these genes (*p* < 0.05 or 0.01, vs. DSS group). Then, the damage to the intestinal barrier was measured. As expected, BO acquired a notable increase in the mRNA expressions of Mucin 2 (MUC2) and Mucin 3 (MUC3) but suppressed the mRNA level of matrix metalloproteinase-9 (MMP-9) in the colon of UC mice (*p* < 0.05 or 0.01, vs. DSS group) (Figure 2D). Further, the immunohistochemical analysis of ZO-1 and Claudin-1 demonstrated that BO could restore the integrity of the intestinal barrier in UC mice (Figure 2E). Similarly, WB assays for ZO-1 and Claudin-1 were consistent with immunohistochemical results (Supplementary Figure S4).

### Modulation of Imbalanced Gut Microbiota in UC Mice by *Bletilla striata* Oligosaccharides

**FIGURE 3:**
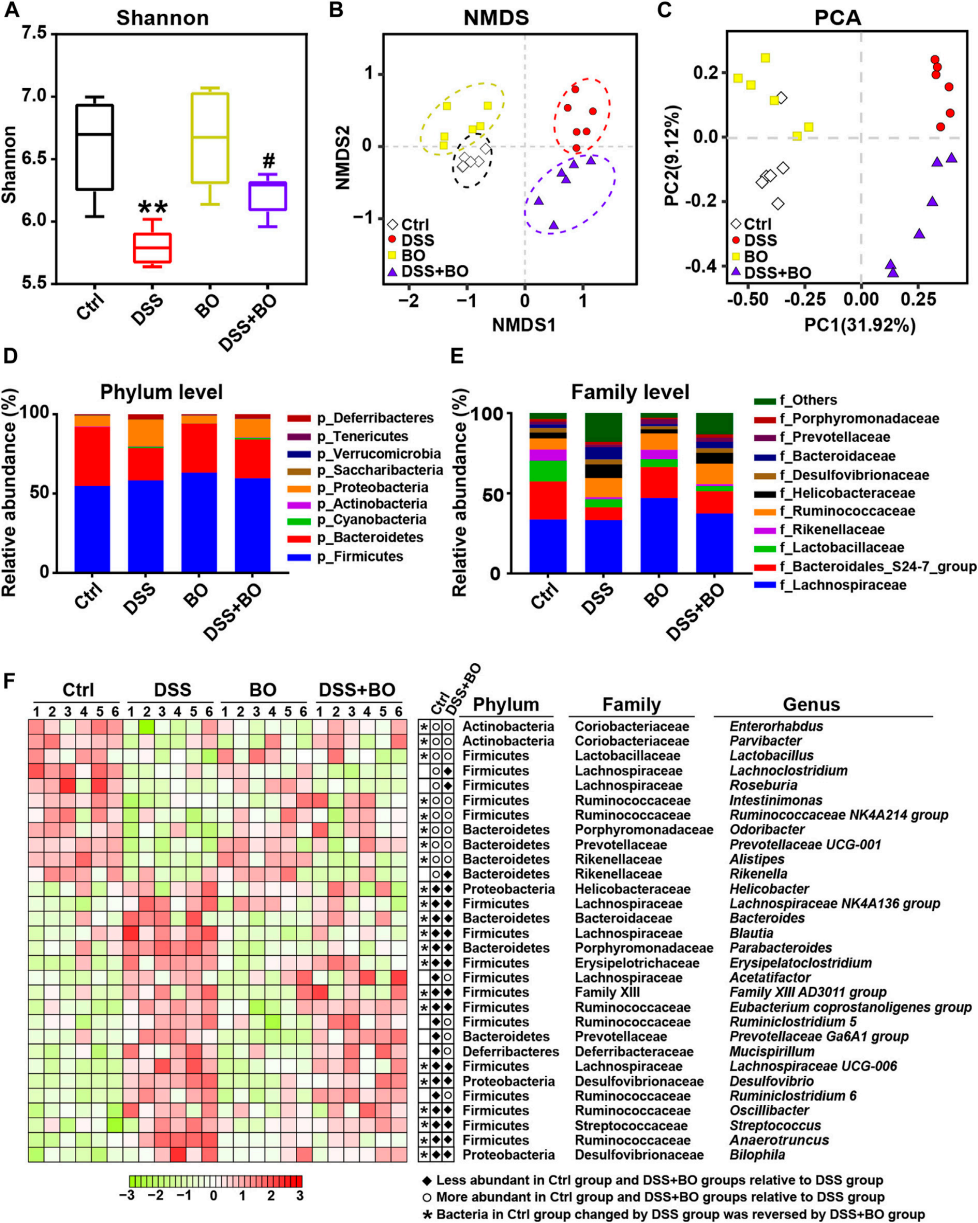
BO alleviated gut dysbiosis in UC mice. **(A)** α-diversity assay calculated with Shannon index. **(B)** Principal component analysis (PCA). **(C)** Non-metric multidimensional scaling (NMDS). **(D)** Taxonomic profiling of gut microbiota at the phylum level. **(E)** Taxonomic profiling of gut microbiota at the family level. **(F)** Variation of 30 OTUs with the greatest changes among four experimental groups at the genus level indicated by a heatmap. Data were shown as means ± SD (*n* = 6). **p < 0.01 vs. Ctrl group; ^#^ *p* < 0.05 vs. DSS group.

Since the occurrence of IBD is closely related to the imbalance of gut microbiota, 16S rDNA sequencing was used to investigate the role of BO in regulating gut microbiota of UC mice. The α-diversity was evaluated by the Shannon index, which was reduced in the DSS group (*p* <0.01, vs. Ctrl group) (Figure 3A), indicating severely damaged richness and diversity of gut microbiota in UC mice. After BO treatment, the reduction of α-diversity was partly restored (*p* <0.01, vs. Ctrl group). Principal component analysis (PCA) and nonmetric multidimensional scaling (NMDS) reflected the β-diversity of gut microbiota among groups. The result shows that four experimental groups were separated entirely into different clusters, suggesting that each group of mice had its unique intestinal bacteria communities (Figures 3B,C).

In our study, Firmicutes, Bacteroidetes, and Proteobacteria are the highest in the contents of gut microbiota in BALB/c mice at the phylum level. In comparison with the Ctrl group, the relative abundance of Bacteroidetes was decreased while Proteobacteria was increased in the DSS group (*p* < 0.05). In contrast, BO intervention effectively reversed the changes of both phyla (*p* < 0.05, vs. DSS group) (Figure 3D). At the family level, BO prevented the decrease in the populations of Rikenellaceae and Bacteroidales S24-7 group, and lowered the abundances of Helicobacteraceae and Bacteroidaceae in UC mice (*p* < 0.05) (Figure 3E). At the genus level, the abundances of 30 OTUs with the most significant changes were shown in a heatmap. Among them, most of the bacteria performed an apparent increase in UC mice like *Parabacteroides*, *Bacteroides*, *Oscillibacter*, and *Helicobacter* (*p* < 0.05 or 0.01, vs. Ctrl group) (Figure 3F; Supplementary Figure S5). Conversely, the contents of other bacteria in the DSS group were lower than those in the Ctrl group, such as Ruminococcaceae *NK4A214 group*, *Odoribacter*, and Prevotellaceae *UCG-001* (*p* < 0.05 or 0.01) (Figure 3F; Supplementary Figure S5). Notably, BO treatment remarkably suppressed the alteration of these imbalanced genera in UC mice (*p* < 0.05 or 0.01, vs. DSS group) (Figure 3F; Supplementary Figure S5).

Next, the Phylogenetic Investigation of Communities by Reconstruction of Unobserved States (PICRUSt) analysis was performed to assess the modulatory effect of BO on metabolic pathways of gut microbiota in UC mice. Based on 147 Keyoto Encyclopedia of Genes and Genomes (KEGG) pathways, 12 evidently changed pathways were chosen for comparisons among four experimental groups. It was suggested that five pathways were downregulated in the DSS group in comparison with those in the Ctrl group, while three pathways were upregulated (Supplementary Figure S6A). On the contrary, three of these altered pathways were recovered to different contents by BO treatment, including Sulfur metabolism, Selenocompound metabolism, Alanine, aspartate, and glutamate metabolism (Supplementary Figure S6B).

### Regulatory Effect of *Bletilla striata* Oligosaccharides on Production of Intestinal Metabolites in UC Mice

**FIGURE 4:**
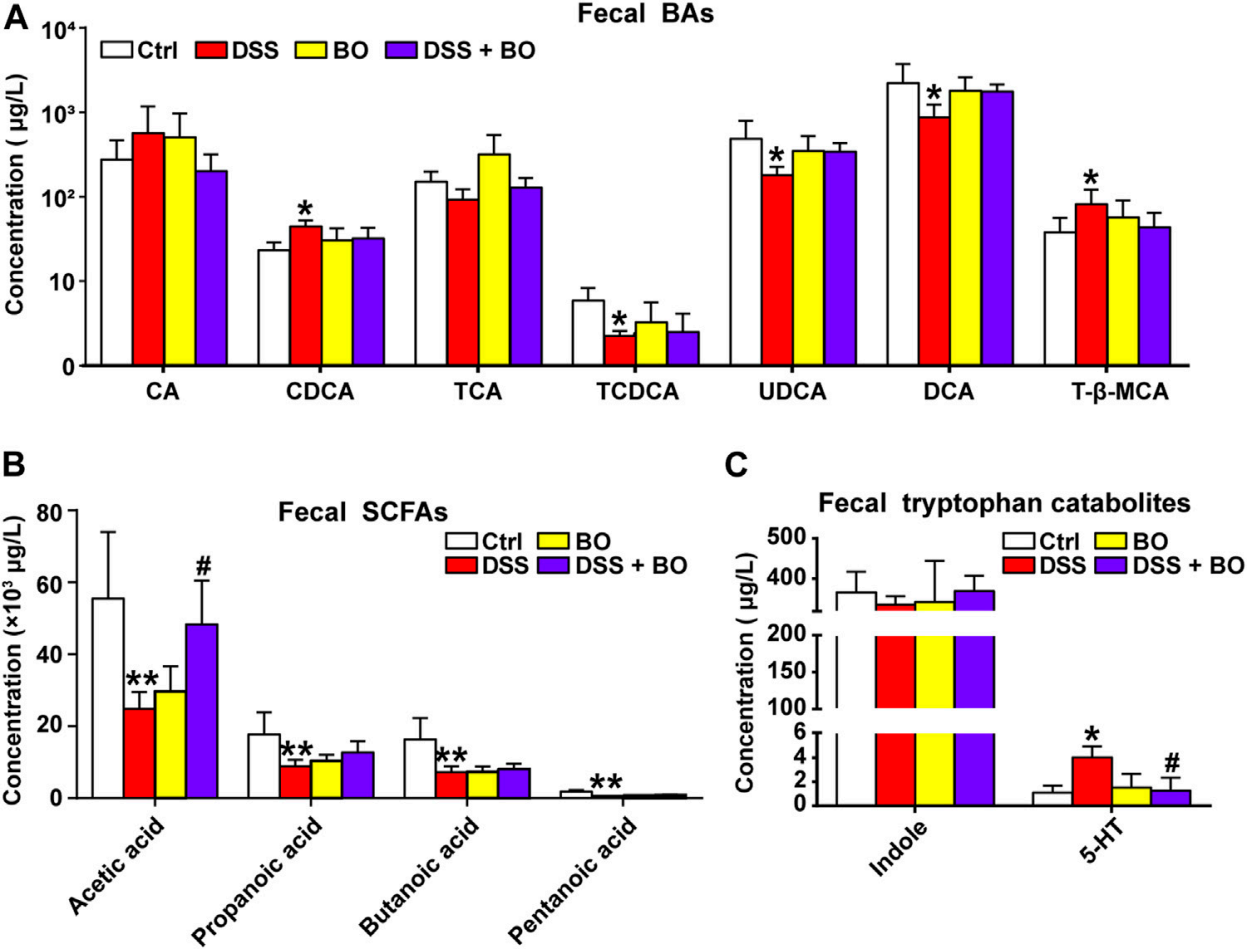
Intervention of BO on intestinal metabolites in feces of UC mice. **(A)** Contents of individual BAs in feces. **(B,C)** Changes of SCFAs. **(B)** and tryptophan catabolites. **(C)** in feces among four experimental groups. Data were shown as mean ± SD (*n* ≥ 3). *p < 0.05, **p < 0.01 vs. Ctrl group; ^#^ *p* < 0.05, ^##^ *p* < 0.01 vs. DSS group.

The altered production of intestinal metabolites usually accompanies the changes in gut microbiota. As indicated in Figure 4A, the levels of CDCA and T-β-MCA were increased in UC mice (*p* < 0.05, vs. Ctrl group), while the productions of TCA, TCDCA, UDCA, and DCA were significantly reduced (*p* < 0.05, vs. Ctrl group). After BO treatment, these altered individual BAs were reversed to certain extents, but no significant difference was captured compared with the DSS group. Compared to the Ctrl group, the contents of four SCFAs in feces of UC mice were considerably reduced (*p* < 0.01), including acetic acid, propanoic acid, butanoic acid, and pentanoic acid. Among them, the amount of acetic acid was profoundly increased after BO administration (*p* < 0.05, vs. DSS group) (Figure 4B). Additionally, we examined tryptophan catabolites (indole and 5-HT). No significant difference of indole was detected among the four experimental groups. However, the level of 5-HT was abnormally increased in UC mice and downregulated after BO treatment (*p* < 0.05, vs. DSS group) (Figure 4C).

### Correlation Between Intestinal Bacterial Abundances and UC-Related Pathological Indices

To explore the relationship between alteration of gut microbiota and UC-related pathological parameters, we calculated the Spearman’s correlation coefficient between four experimental groups as indicated in Supplementary Figure S8. It was found that most of the changed intestinal bacteria at genus levels were positively or negatively correlated with physiochemical parameters, inflammatory responses, intestinal barrier integrity, and bacterial metabolism, suggesting the pivotal role of gut microbiota in the occurrence of UC.

### Effects of *Bletilla striata* Oligosaccharides and *Bletilla striata* Oligosaccharides Fermentation Broth on DSS-Induced UC in Mice With Gut Microbiota Depletion

**FIGURE 5:**
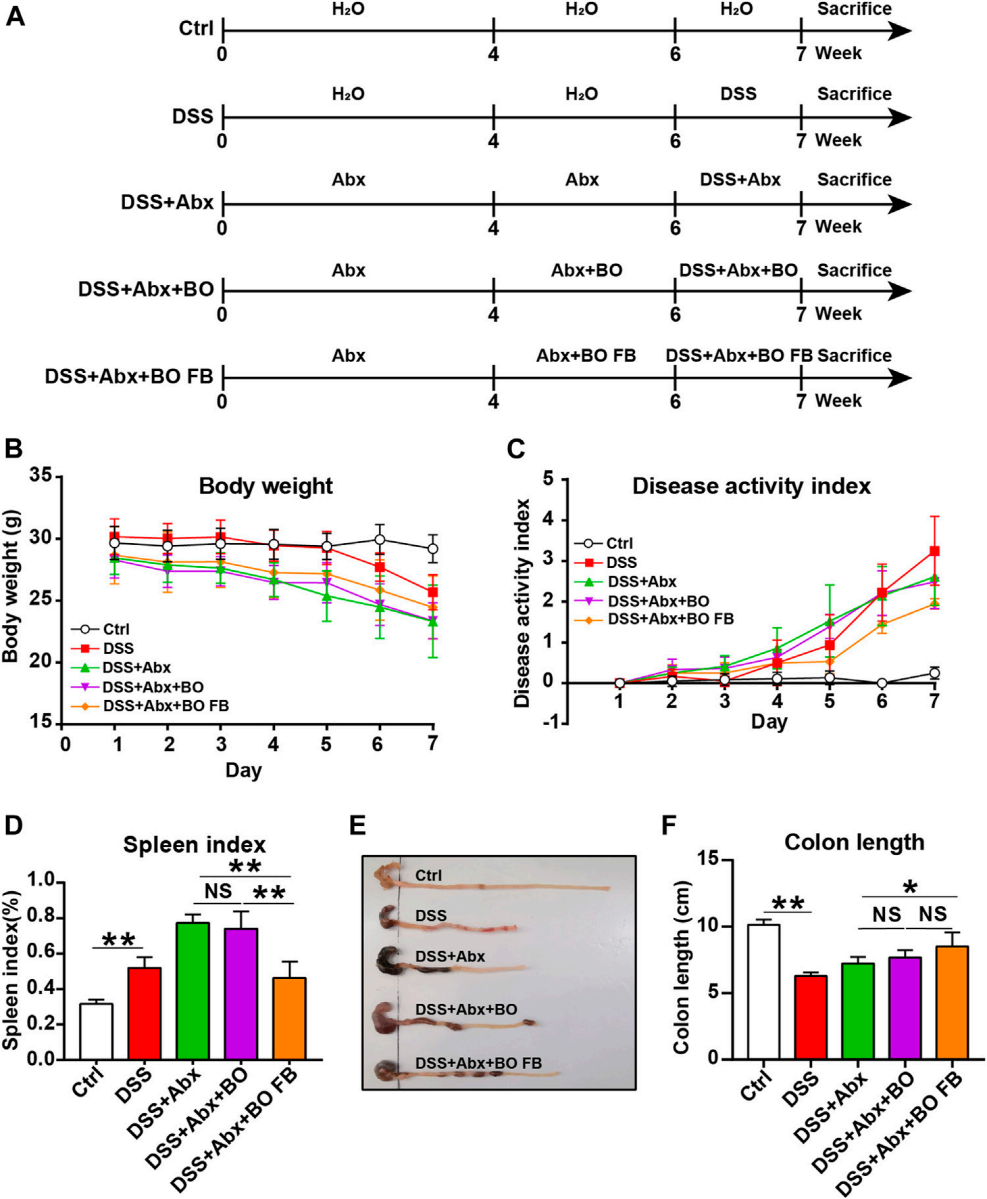
Effect of BO and BO fermentation broth on physiological indices of UC mice with depleted gut microbiota. **(A)** BALB/c mice were treated with Abx (ampicillin, 1 mg/ml; vancomycin, 0.5 mg/ml; neomycin sulfate, 1 mg/ml; metronidazole, 0.5 mg/ml) for 4 weeks, followed by the treatment with DSS, BO, or BO fermentation broth. **(B)** Body weight. **(C)** DAI scores. **(D)** Spleen index. **(E)** Photographs of the colon. **(F)** Colon length. Data were shown as means ± SD (*n* = 6). *p < 0.05, **p < 0.01.

**FIGURE 6:**
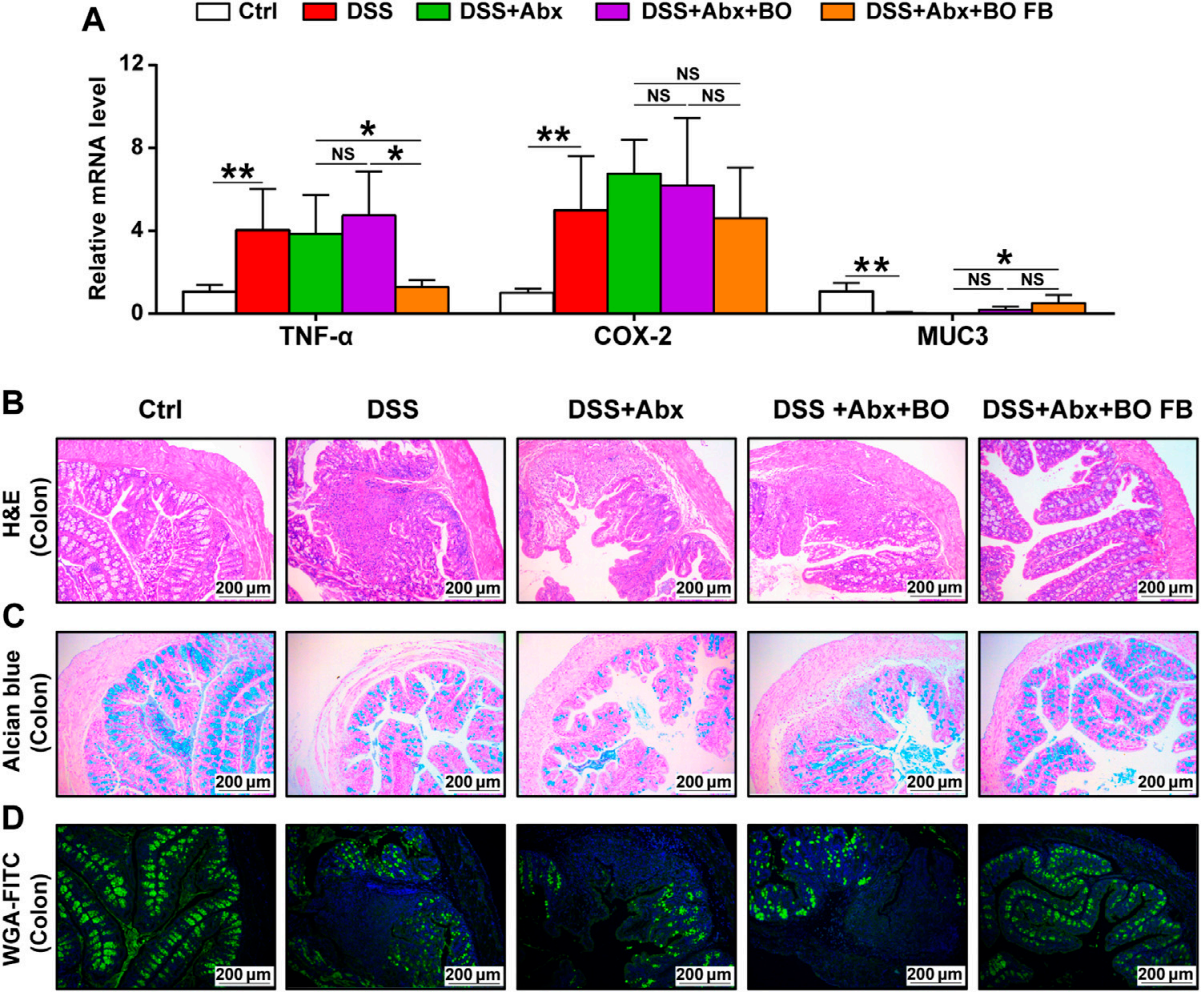
Effect of BO and BO fermentation broth on intestinal inflammation and gut barrier damage in UC mice with depleted gut microbiota. **(A)** Expressions of TNF-α, COX-2, and MUC3 at mRNA levels. **(B)** Morphological analysis of the colon stained by Hematoxylin & eosin (H&E). Scale bar = 200 μm. **(C)** Morphological analysis of the colon stained by Alcian blue. Scale bar = 200 μm. **(D)** Morphological analysis of the colon stained by wheat germ agglutinin labeled with FITC. Scale bar = 200 μm. Data were shown as mean ± SD (*n* = 6). *p < 0.05, **p < 0.01.

To confirm the significance of BO in the protection against UC, we treated mice with Abx for 4 weeks to deplete the gut microbiota (Figure 5A). As shown in Supplementary Figure S7, the quantification of intestinal bacteria by RT-qPCR proved that Abx entirely destroyed the gut microbiota of mice. In addition, compared to the DSS group, the physiological indices, inflammatory responses, and gut barrier damage were not improved or even worsened in mice from the DSS + Abx group or DSS + Abx + BO group (Figures 5, 6). On the contrary, BO fermentation broth (BO FB) significantly suppressed the alteration of physiological indices in UC mice with gut microbiota depletion, such as the weight loss (Figure 5B), DAI scores (Figure 5C), spleen index (Figure 5D), and colon length (Figures 5E,F) (*p* < 0.01 or 0.05, vs. DSS + Abx group). Moreover, BO FB considerably reversed the upregulation of inflammatory cytokines (TNF-α and COX-2) and reduction of MUC3 in the colon (Figure 6A), and damage to the gut barrier (Figures 6B–D) in gut microbiota-depleted UC mice. At the protein level, BO FB not only promoted the expression of ZO-1 and Claudin-1 but also inhibited the expression of NLRP3 and p-ERK (Supplementary Figure S9).

## Discussion

Accumulating evidence shows that the symptoms of DSS-induced UC mice were similar to those of UC patients. In the present study, UC mice were characterized by diarrhea, abdominal pain, and bloody stools, consistent with a previous report (Kim et al., 2012). However, after BO treatment, the severity of bloody stools was lessened with the reinstated colon injury and reduced spleen weight in UC mice, indicating the preventive effect of BO on the occurrence of UC (Figures 1A–E). Moreover, BO effectively suppressed DSS-induced damage to the intestinal tract in UC mice (Figures 1F–H), parallel to our previous studies, in which BO were found to avoid the destruction of gut barrier in obese mice by the regulation of gut microbiota (Hu et al., 2020).

NLRP3 inflammasome is widely distributed in epithelial and immune cells, where NLRP3 can activate the MAPK signaling pathways and further lead to the secretion of proinflammatory cytokines in UC (Zhen & Zhang, 2019). MAPKs are a group of cytoplasmic enzymes that mediate the transmission of inflammatory signals from the cell membrane to the nucleus. Upon activation, MAPKs can phosphorylate the serine/threonine amino acids of downstream intracellular proteins, which further initiate the subsequent cascade reactions (Hommes et al., 2003). In this study, BO repressed the phosphorylation of ERK and JNK (members of MAPK family) by inactivating the NLRP3 inflammasome (Figures 2A–C). In addition, TNF-α not only mediates the expression of IL-6 and IL-1β but also acts as a key binding site for the NF-κB pathway that is vital to the pathogenesis of UC (Sands & Kaplan, 2007). Here, the over-expressions of three cytokines were significantly inhibited by BO treatment (Figure 2A), suggesting that BO may improve UC by downregulating the *in vivo* inflammation. Besides, BO reduced the expressions of COX-2 and iNOS in the colon of UC mice (Figure 2A). COX-2 was reported to affect intestinal epithelial regeneration and induce the pro-inflammatory response initiated by TNF-α (Li et al., 2018), and iNOS directly caused intestinal damage by promoting the production of NO (Gochman et al., 2012). Both kinases are also inducers of cellular stress, indicative of a potential of BO for ameliorating oxidative injury in the development of UC.

Diarrhea is one of the most apparent symptoms of UC, and its pathogenesis may attribute to the sustained and diffuse inflammation that increases the risk of intestinal mucosal damage and finally cause the dysfunction of ion transporters and channels in intestinal epithelia (Anbazhagan et al., 2018). In this study, the mRNA levels of AQP2, AQP3, and ENaC-β were statistically increased in UC mice after BO treatment (Figure 2D). We presume that BO might alleviate diarrhea by strengthening the absorption of Na^+^ and water in the intestinal lumen of UC mice. On the other hand, the mucus protein secreted by goblet cells forms a mucus layer, which covers the intestinal mucosa surface and constitutes the first defense line of the gut barrier (Schoultz & Keita, 2019). In addition, the interaction of TJ proteins like Occludin, Claudin-1, and ZO-1 controls intestinal epithelial permeability under physiological state (Konig et al., 2016), while DSS administration may directly cause the loss of goblet cells and destruction of TJ proteins, ultimately resulting in the infiltration of intestinal pathogens (Landy et al., 2016). Interestingly, BO significantly increased the expression of glycoprotein mucins and TJ proteins (Figures 2D,E). Based on these findings, we proposed the effectiveness of BO on repairing intestinal barrier damage.

Once the intestinal mucus layer is disrupted, harmful bacteria can easily penetrate intestinal epithelia and lead to changes in gut microbial composition (Pei et al., 2019). In our study, a significant decrease in α-diversity was observed in UC mice, which meant the reduction of species diversity of intestinal flora in the occurrence of UC (Figure 3A). The abundance analysis of gut microbiota at phylum, family, and genus levels further revealed the variability of intestinal florae between the DSS group and the DSS + BO group (Figures 3D–F). *Helicobacter*, *Desulfovibrio*, and *Oscillibacter* are among these most changed genera. *Helicobacter* can induce the activation of pathogenic T cells, disrupt intestinal immune function, and finally promotes UC development (Chow et al., 2011; Xu et al., 2018). *Desulfovibrio* is one of the major sulfate-reducing bacteria in the intestine of humans and contributes to the generation of H_2_S that is toxic to intestinal epithelia and may induce cellular apoptosis (Rowan et al., 2010). *Oscillibacter* can worsen intestinal permeability and will be obviously elevated after DSS administration (Wu et al., 2019). The abundance of this bacterial is also positively correlated with the levels of pro-inflammatory cytokines such as IL-6 and IL-1β (Wu et al., 2019). Furthermore, *Oscillibacter* was found to be relevant to the expressions of NLRP3 and TNF-α in our study (Supplementary Figure S8). The aberrant proliferation of these bacteria is detrimental to the intestinal structure and will exacerbate the development of UC. Expectedly, their abundances were decreased in UC mice after BO treatment (Figure 3F; Supplementary Figure S5). Further, by PICRUSt analysis, BO were found to revert the altered metabolic pathways of gut microbiota in UC mice, implicating its ability to regulate the metabolism of flora (Supplementary Figure S6).

Interestingly, several intestinal bacteria that metabolize BAs were also changed in UC mice, such as *Bacteroides*, *Ruminococcaceae*, and *Lactobacillus* (Figure 3F; Supplementary Figure S5). It was reported that conjugated BAs (TCA and TCDCA) could be hydrolyzed into primary BAs (CA and CDCA) by *Bacteroides* through bile salt hydrolase (BSH) in the intestine (Jia et al., 2018). Then, CA and CDCA were converted into secondary BAs (DCA and LCA) *via* the 7α dehydroxylation of *Ruminococcaceae* (Camilleri, 2015). In this study, the sequencing of gut microbiota revealed an increased *Bacteroides* and a decreased *Ruminococcaceae* in UC mice, which led to a decrement of secondary BAs (Figure 4A). A similar result was illustrated in recent studies (Dong et al., 2021; Gao et al., 2021; Sinha et al., 2020). For example, the lack of secondary BAs like DCA and UDCA may aggravate intestinal inflammation due to the inhibition of immune regulators. On the other hand, the decrease of beneficial bacteria *Lactobacillus* will cause the accumulation of T-β-MCA in the intestine, an antagonist of FXR, and thus interrupts the activation of intestinal FXR signaling (Li et al., 2013). In the present study, BO restored the abundance changes of *Bacteroides* and Ruminococcaceae, and partly ameliorated the disturbance of BA metabolism (Figure 4A), suggesting a potential modulatory effect on the production of secondary BAs in UC mice. Besides, SCFAs can provide energy to colon cells, block the NLRP3 inflammasome activation, protect the intestinal barrier integrity, and prevent the deterioration of UC (Feng et al., 2018). Here, we found that the levels of four SCFAs were statistically reduced in feces of the DSS group, accompanied by the decrease of SCFA-producing bacteria like *Odoribacter* and *Intestinimonas* (Figure 3F; Supplementary Figure S5). Conversely, BO increased the abundances of both bacteria with the high acetic acid content, which could enhance epithelia-mediated intestinal defense function (Fukuda et al., 2011). In addition, BO suppressed the production of tryptophan metabolite (5-HT) in the DSS group (Figure 4C). The harmful effect of 5-HT on UC was attributed to the stimulation of colonic leukocyte recruitment and overexpression of NADPH oxidase (Dong et al., 2019). By correlation analysis, these changed intestinal bacteria were further proved to be associated with the abnormality of their metabolites (Supplementary Figure S8). Thus, BO may prevent the development of UC by modulating the imbalance of gut microbiota and the alteration of intestinal metabolites.

It is difficult for complex carbohydrates to be absorbed in the intestinal tract, but they can be metabolized by gut microbiota as the substrates and then exert indirect biological activities. Indeed, this was confirmed in our antibiotic experiments, in which it was BO fermentation broth rather than BO that improved the physiochemical indices in UC mice with depleted gut microbiota (Figures 5, 6). It points toward the necessity of gut microbiota in mediating the metabolism of BO and subsequent prevention of UC.

## Conclusion

**FIGURE 7:**
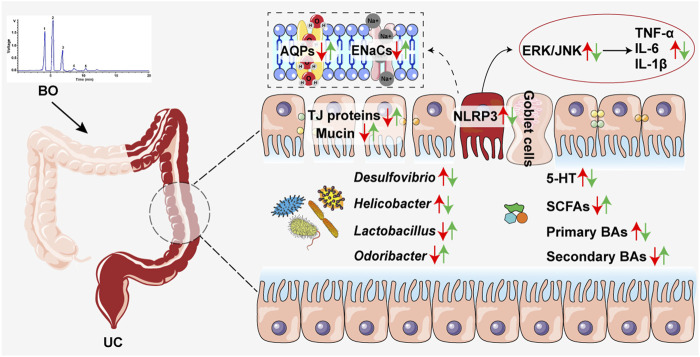
Schematic diagram showing how BO improved UC. Red arrows, increased or decreased changes between the Ctrl group and the DSS group. Green arrows, increased or decreased changes between the DSS and the DSS + BO groups.

Taken together, this study demonstrated that BO displayed a preventive effect on UC, accompanied by the suppression of intestinal inflammation and gut barrier damage. The underlying therapeutic mechanisms were associated with inhibiting gut microbiota dysbiosis and reversing abnormal production of intestinal metabolites (Figure 7). Overall, our findings revealed a potential application of BO in the treatment of UC in the future.

## Data Availability

The datasets presented in this study can be found in online repositories. The names of the repository/repositories and accession number(s) can be found below: National Center for Biotechnology Information (NCBI) BioProject database under accession number PRJNA787415.

## Glossary

5-HT: 5-hydroxytryptamine
Abx: antibiotic mixtures
AQP: aquaporin
BAs: bile acids
BO: *Bletilla striata* oligosaccharide
BP: *Bletilla striata* polysaccharide
CA: cholic acid
CDCA: chenodeoxycholic acid
COX-2: cyclooxygenase-2
DAI: disease activity index
DCA: deoxycholic acid
DSS: dextran sulfate sodium
ENaC: epithelial sodium channel
ERK1/2: extracellular regulated kinase 1/2
IBD: inflammatory bowel disease
IL: interleukin
iNOS: inducible nitric oxide synthase
JNK: c-Jun N-terminal kinase
LC/GC-MS: liquid chromatography/gas chromatography-mass spectrometer
Lcn2: Lipocalin-2
MMP-9: matrix metalloproteinase-9
MUC: mucin
NLRP3: NOD-like receptor pyrin domain containing 3
SCFAs: Short-chain fatty acids
TCA: taurocholic acid
TCDCA: taurochenodeoxycholic acid
TNF-α: tumor necrosis factor-α
T-β-MCA: tauro-β-murocholic acid
UC: ulcerative colitis
UDCA: ursodeoxycholic acid
ZO-1: zonula occludens-1
