# Supplementary material for: Bletilla striata Oligosaccharides Improve Ulcerative Colitis by Regulating Gut Microbiota and Intestinal Metabolites in Dextran Sulfate Sodium-Induced Mice
Source: Front Pharmacol. 2022 Apr 25;13:867525. doi: 10.3389/fphar.2022.867525 (PMC9081565; doi:10.3389/fphar.2022.867525)
Supplement: Supplementary file 1 [file DataSheet1.docx]

# Supplementary Data

**Supplementary Methods**

1. Monosaccharide composition analysis of BO.
2. Identification of polymerization degree of BO.
3. Gas Chromatography-Mass Spectrometry (GC-MS) analysis on SCFAs and indole.
4. Liquid Chromatography-Mass Spectrometry (LC-MS) analysis on 5-hydroxytryptamine (5-HT).
5. Extraction and quantification of fecal bacterial genomic DNA.

**Supplementary Figures**

Fig. 1. Analysis on monosaccharide composition of BO by HPLC.

Fig. 2. Structural analysis of BO.

Fig. 3. Serum level of the inflammatory marker Lcn2 in different treatment group

Fig. 4. Protein expressions of ZO-1 and Claudin-1 in colon tissues by WB.

Fig. 5. Regulatory effect of BO on imbalanced gut microbiota in UC mice at the genus level.

Fig. 6. Effect of BO treatment on metabolic pathways of gut microbiota in UC mice by PICRUSt analysis.

Fig. 7. Quantification of intestinal bacteria in mice with/without gut microbiota depletion.

Fig. 8. Spearman’s correlation analysis between gut microbiota at genus levels with significant changes and UC-related parameters.

Fig. 9. The representative images of Western blotting analysis of ZO-1, Claudin-1, NLRP3, p-ERK, and ERK in UC mice with depleted gut microbiota.

**Supplementary Tables**

Table 1. Criteria for scoring disease activity index.

Table 2. Composition of simulated intestinal environment medium.

Table 3. List of primer sequences for RT-qPCR analysis of fecal samples.

Table 4. List of primer sequences for RT-qPCR analysis of major tissues.

## Supplementary Methods

### Monosaccharide composition analysis of BO

*B. striata* oligosaccharides (BO) were degraded and derived using PMP for monosaccharide composition analysis as previously. Briefly, 5 mg of BO were hydrolyzed in 3 mL of trifluoroacetic acid (2 M) at 120 °C for 2 h. After the removal of residual trifluoroacetic acid, 600 μl of NaOH (0.3 M) and 600 μl of PMP (0.3 M) solution were added and reacted at 70 °C for 30 min. Finally, the solution was neutralized to with hydrochloric acid (0.3 M), followed by multiple extractions with chloroform to remove the impurities. The aqueous layer solution was filtered through a 0.45 μm of filter and ready for HPLC analysis. The monosaccharide standards, i.e., mannose and glucose, were derived as the same to BO samples.

The chromatographic analysis on monosaccharide composition of BO was performed using Agilent 1260 HPLC system coupled with a Uranus 5u C18 column (4.6 mm × 150 mm, 5 μm) (FLM Scientific Instrument Co. Ltd, Guangzhou, China) and detected by ultraviolet detector. The column temperature was set as 40 ºC. The mobile phase was composed of acetonitrile (A) and ammonium acetate (20 mM) (B). The gradient elution was performed as follows: 0-5 min, 16 % A; 5-17 min, 16% to 19% A; 17-30 min, 19% to 22% A; 30-35 min, 22% to 16% A. The injection volume was 10 μl and the flow rate of mobile phase was 0.3 mL/min.

### Identification of polymerization degree of BO

To identify the polymerization degree of BO, HPLC-MS analysis was performed on an ultra-high performance liquid chromatography system (SCIEX, Framingham, MA, USA) with an Agilent Poroshell Hilic colum (2.1 × 100 mm, 2.7 μm) (Santa Clara, CA, USA) fitted with a Triple TOF 4600 high resolution mass spectrometry system (SCIEX, Framingham, MA, USA). The mobile phase was composed of 0.1% aqueous formic acid (A) and acetonitrile (B). The gradient elution was performed as follows: 0-1 min, 100% B; 1-2 min, 100% to 85% B; 2-32 min, 85% to 50% B; 32-33 min, 50% to 20% B; 33-37 min, 20% to 85% B; 37-44 min, 85% B. The injection volume was 10 μl and the flow rate was 0.03 ml/min. The ESI source in positive ion mode was used with resolving power 30,000 and a scan range of m/z 100-1000. The parameters were set as followed: capillary temperature, 600 °C; ion spray voltage, 5.5 kV in positive ion mode; sheath and auxiliary gas flow rate, 55 psi; collision energy, 35 eV.

### Gas Chromatography-Mass Spectrometry (GC-MS) analysis on SCFAs and indole

SCFAs and indole were detected by Agilent GC/MS 5975 (Agilent, CA, USA) equipped with a DB-WAX column (30 m × 0.32 mm × 0.5 μm) (Agilent, CA, USA). The oven temperature of column was programmed as following heating procedure: the initial temperature was kept at 50 °C for 1 min, then raised 10 °C/min to 250 °C and maintained for 2 min with high purity helium (purity > 99.999%) as carrier gas (flow rate: 1.0 mL/min). Fecal samples were injected at a diffluent rate of 1:50 accompanied by an electron impact source. The electron energy was 70 eV. The ion source temperature and interface temperature were set as 230 °C and 250 °C, respectively. The solvent delay time was set to 6 min. Acetic acid, propinoic acid, butyric acid, valeric acid, and indole were used as standard references.

### LC-MS analysis on 5-hydroxytryptamine (5-HT)

The 5-HT were analyzed by Agilent 6460 quadrupole series mass spectrometer (Agilent, CA, USA) equipped with a ZORBAX SB-C18 column (2.1 × 50 mm) (Agilent, CA, USA). The mobile phase was composed of both 0.1% aqueous acetic acid solution (A) and acetonitrile (B). And the gradient elution program was shown as below: 0-1 min, 5% B; 1-2 min, 5% to 65% B; 2-4 min, 65% to 90% B; 4-6.5 min, 90% to 95% B; 6.5-8 min, 95% to 0% B. The volume of injection was 10 μL and the flow rate of mobile phase was 0.2 mL/min. The electrospray ionization source in positive ion pattern was used in MS analysis, accompanied by the following parameters: capillary voltage, 3.5 kV; temperature and flow rate of sheath gas, 400 °C and 600 L/h. The 5-HT were considered as standards.

### Extraction and quantification of fecal bacterial genomic DNA

Fecal samples (25 mg) were homogenized with 1% sterile PBS until the feces became mushy, followed by centrifugation at 3000 rpm for 5 min. The upper bacterial solution was treated with proteinase K, RNase, and lysozyme in a shaker at 37 °C for one hour. After the addition of lysate and DNA extraction agent, the bacterial solution was incubated in a water bath at 65 °C for 10 min. Then, the solution was centrifuged at 13,000 rpm for 10 min, and the supernatant was collected to precipitate DNA using isopropyl alcohol. After centrifugation, the DNA precipitation was washed with 75% ethanol and dissolved by sterile water after drying at room temperature. The bacterial DNA was quantified on a CFX Connect Real-time system (Bio-Rad, Hercules, CA, USA) by using 2 × FastHS SYBR QPCR mixture (AllMEEK, Beijing, China) with the following thermal cycle condition: initial at 95 °C for 10 min, 34 cycles of amplification (30 s at 95 °C for denaturation, 30 s at 58 °C or 55 °C for annealing, 30 s at 72 °C for extension), terminal extension at 72 °C for 5 min. Five standard bacteria, i.e., *Bacteroides*, *Enterococcus*, *Akkermansia*, *Bifidobacterium*, and *Salmonella*, were used to represent Bacteroidetes, Firmicutes, Verrucomicrobia, Actinobacteria, and Proteobacteria phyla, respectively. The primer sequences of target intestinal bacteria were listed in Supplementary Table 2. Bacterial contents were quantified by the standard curves using corresponding bacteria plasmids with gradient dilution.

## Supplementary Figures


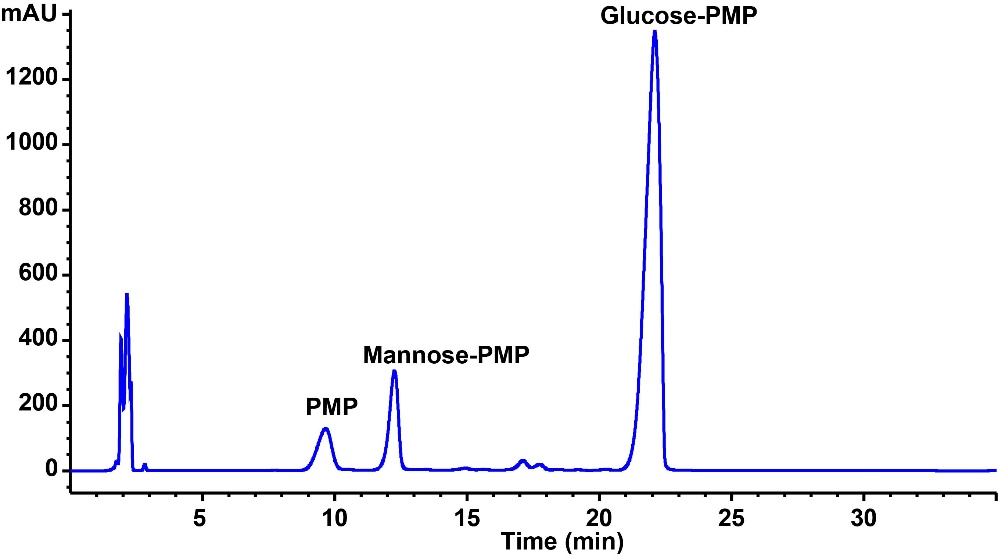


Fig. 1. Analysis on monosaccharide composition of BO by HPLC. After the hydrolysis by trifluoroacetic acid, BO were derivatized with PMP, followed by HPLC assay.


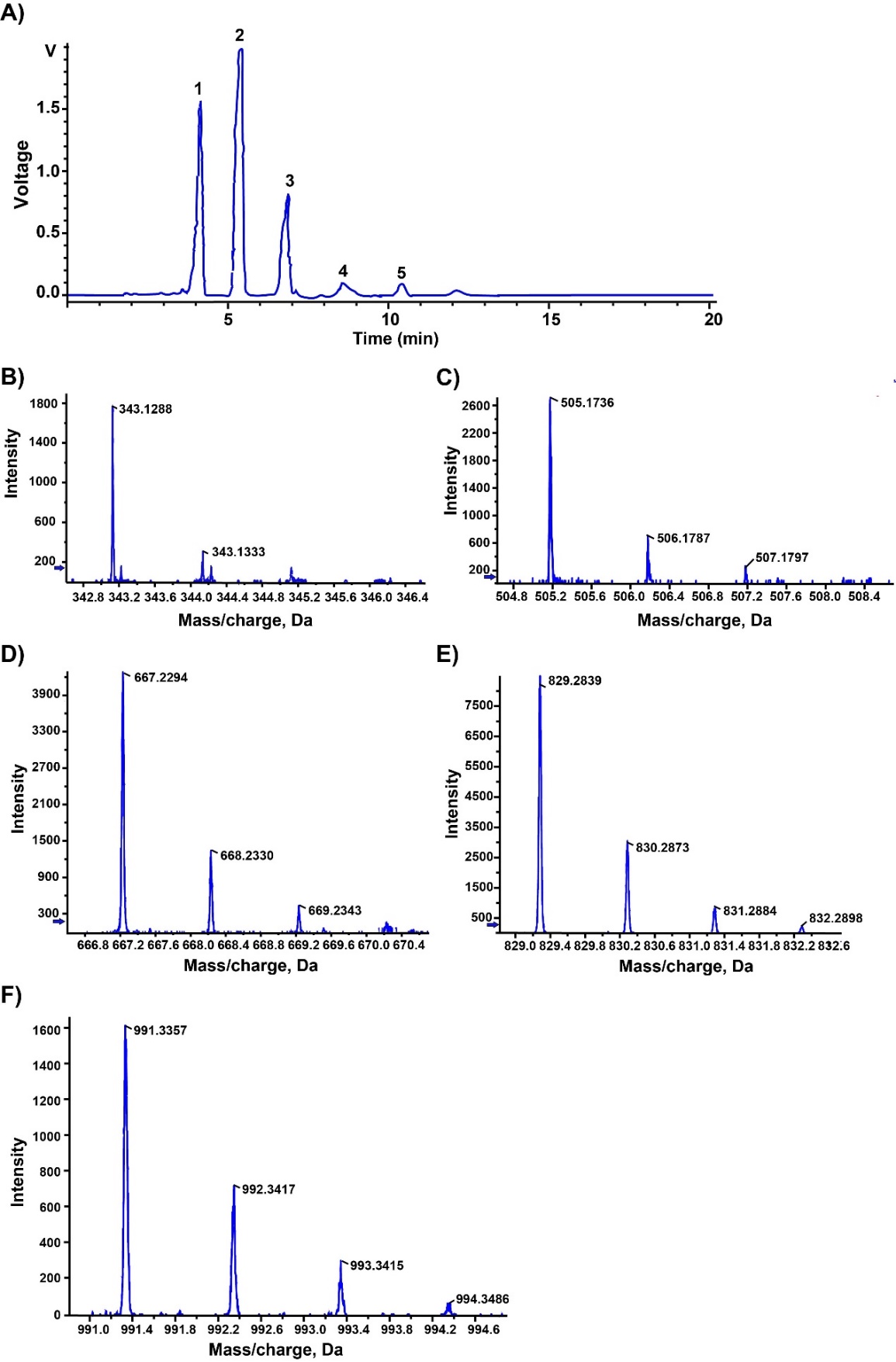


Fig. 2. Structural analysis of BO. (A) HPLC of BO. (B) Disaccharide mass spectrogram of BO. (C) Trisaccharide mass spectrogram of BO. (D) Tetrasccharide mass spectrogram of BO. (E) Pentasaccharide mass spectrogram of BO. (F) Hexasaccharide mass spectrogram of BO. 1, Glucose dimerization; 2, Glucose trimerization; 3, Glucose tetramer; 4, Glucose pentamerization; 5, Glucose hexamer.


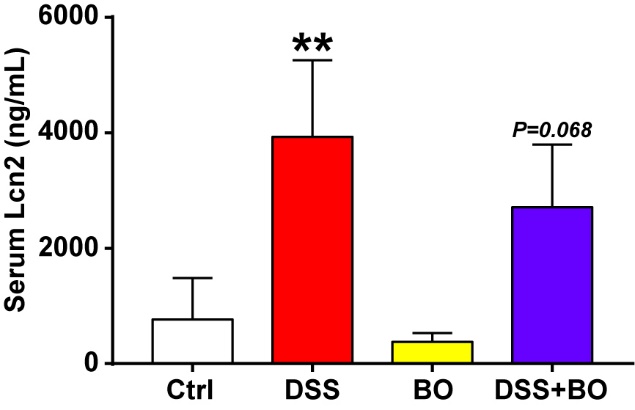


Fig. 3. Serum level of the inflammatory marker Lcn2 among experimental groups. Data were shown as means ± SD (n = 6). ^**^*P* < 0.01 *vs* Ctrl group.


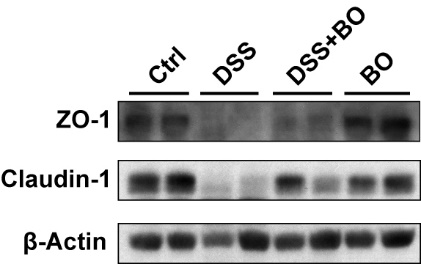


Fig. 4. Protein expressions of ZO-1 and Claudin-1 in colon tissues by Western blot.


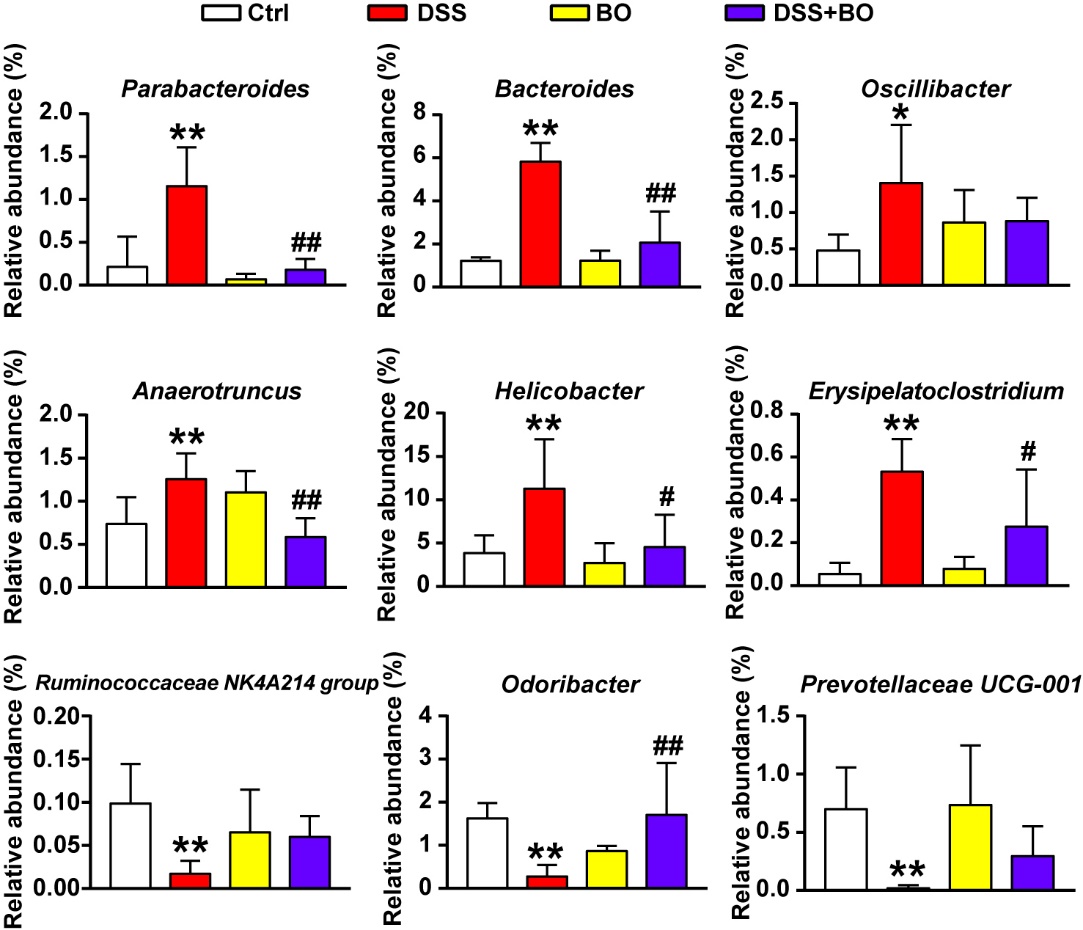


Fig. 5. Regulatory effect of BO on imbalanced gut microbiota in UC mice at the genus level. Data were shown as means ± SD (n = 5−8). ^*^*P* < 0.05, ^**^*P* < 0.01, *vs.* Ctrl group; ^#^*P* < 0.05, ^##^*P* < 0.01, *vs.* DSS group.


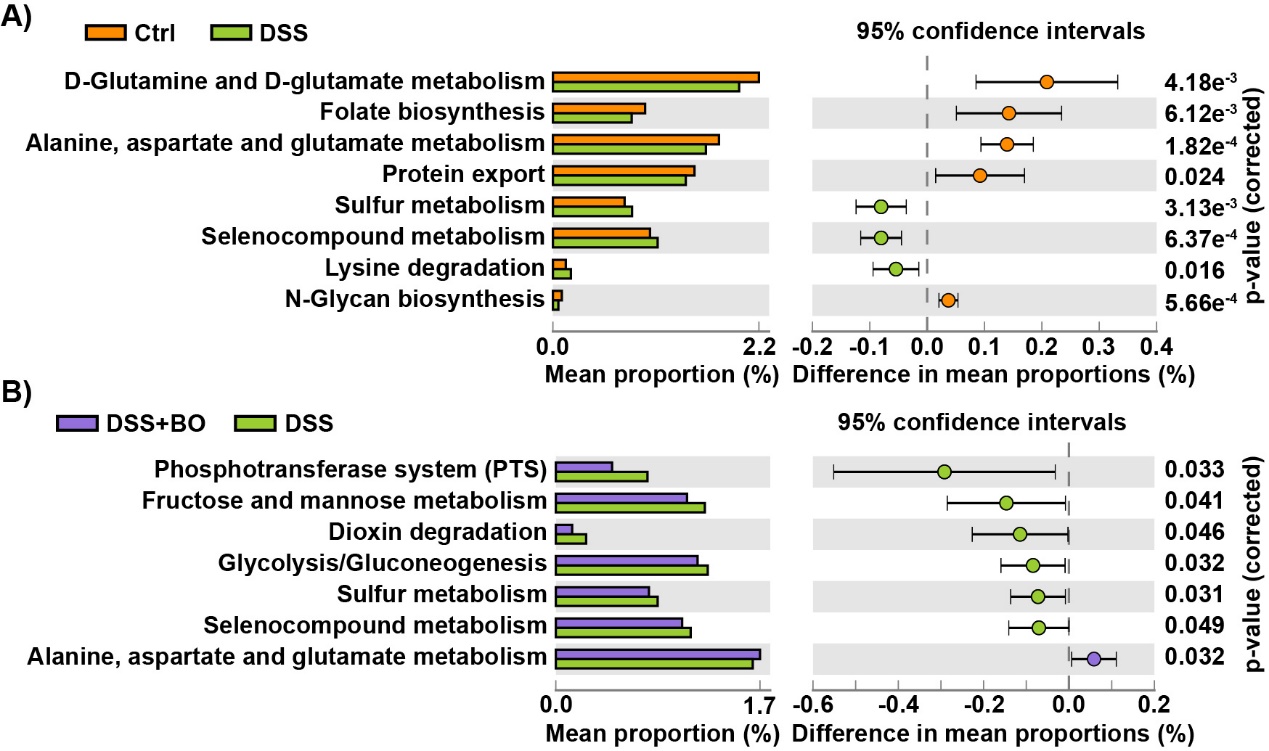


Fig. 6. Effect of BO treatment on metabolic pathways of gut microbiota in UC mice by PICRUSt analysis. All metabolic pathways were screened based on the KEGG database. (A) Comparison of significantly changed eight pathways between the Ctrl group and the DSS group. (B) Comparison of significantly changed seven pathways between the DSS group and the DSS + BO group.


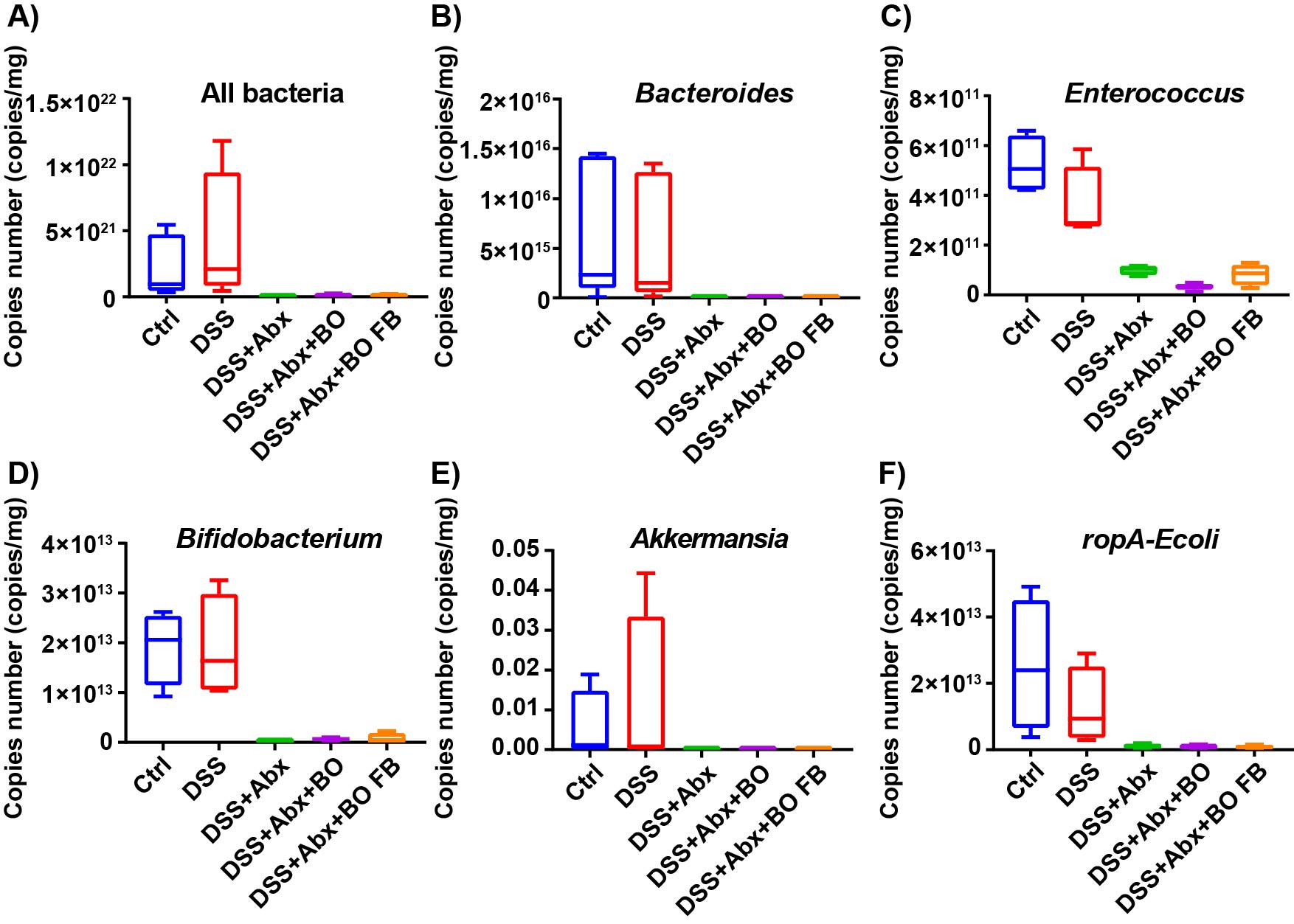


Fig. 7. Quantification of intestinal bacteria in mice with/without gut microbiota depletion. (A) Content of All bacteria in fecal samples of mice by RT-qPCR. (B−F) Phylum abundances of gut microbiota by RT-qPCR, in which *Bacteroides* (B), *Enterococcus* (C), *Bifidobacterium* (D), *Akkermansia* (E), and *Salmonella* (F), were used as representative bacteria of Bacteroidetes, Firmicutes, Verrucomicrobia, Actinobacteria, and Proteobacteria phyla, respectively.


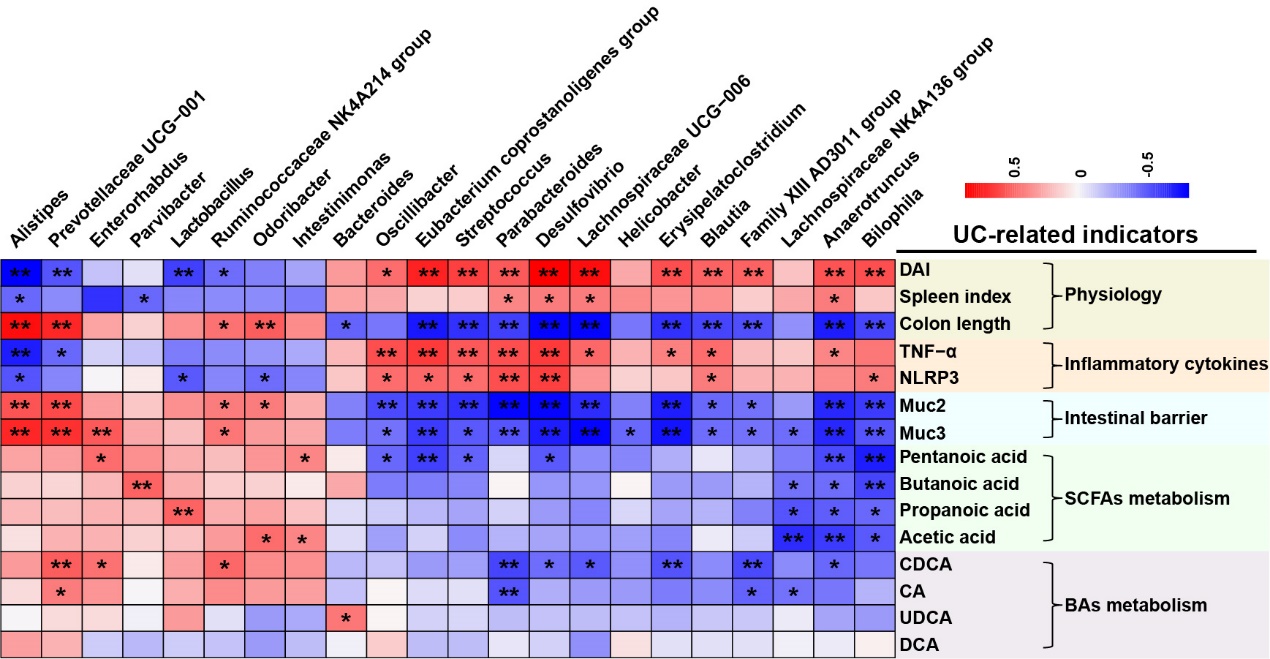


Fig. 8. Spearman’s correlation analysis between gut microbiota at genus levels with significant changes and UC-related parameters. ^*^*P* < 0.05, ^**^*P* < 0.01.


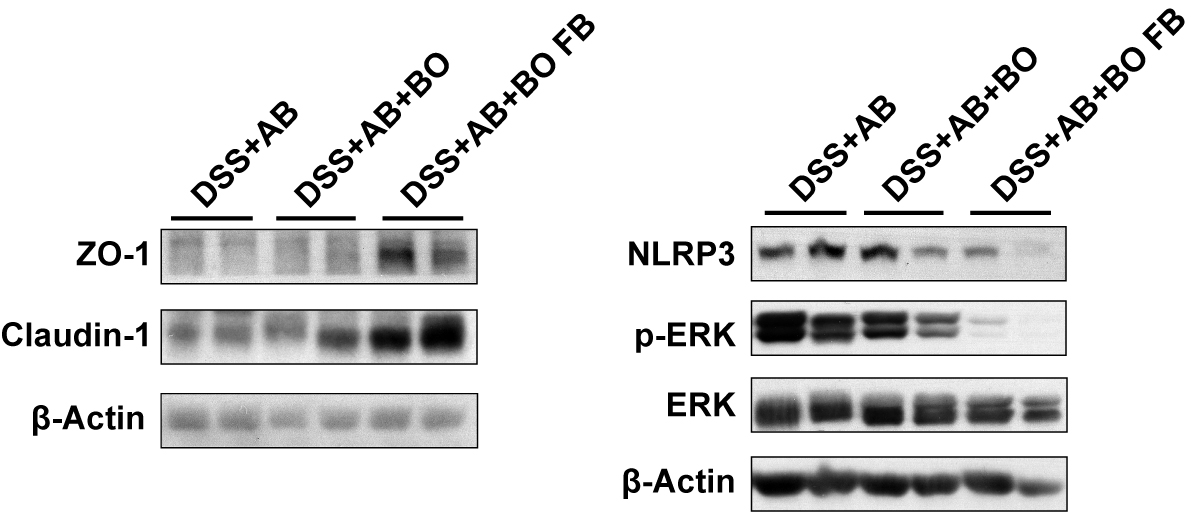


Fig. 9. Protein levels of ZO-1, Claudin-1, NLRP3, p-ERK, and ERK in colon tissues of UC mice with depleted gut microbiota by Western blot.

## Supplementary Tables

Table 1. Criteria for scoring disease activity index

| Score | Weight loss (%) | Stool consistency | Gross bleeding |
| --- | --- | --- | --- |
| 0 | None | Normal | Negative |
| 1 | 1−5 | Loose stool | Negative |
| 2 | 5−10 | Loose stool | Hemoccult positive |
| 3 | 10−15 | Diarrhea | Hemoccult positive |
| 4 | >15 | Diarrhea | Gross bleeding |

Table 2. Composition of simulated intestinal environment medium

| Component | Content (g/L) |
| --- | --- |
| K_2_HPO_4_ | 0.293 |
| KH_2_PO_4_ | 0.176 |
| NaCl | 0.443 |
| (NH_4_)_2_SO_4_ | 0.450 |
| CaCl_2_ | 0.045 |
| MgCl_2_·6H_2_O | 0.093 |
| L-cysteine | 0.500 |
| ascorbic acid | 0.500 |
| Na_2_CO_3_ | 4.000 |
| [tryptone](javascript:;) | 1.000 |
| [yeast](javascript:;) [extract](javascript:;) | 1.000 |

Table 3. List of primer sequences for RT-qPCR analysis of fecal samples

| Name | Forward primer (5'-3') | Reverse primer (5'-3') |
| --- | --- | --- |
| All bacteria | ACTCCTACGGGAGGCAGCAGT | ATTACCGCGGCTGCTGGC |
| Bacteroides | GGTTCTGAGAGGAGGTCCC | GCTGCCTCCCGTAGGAGT |
| Enterococcus | CCCTTATTGTTAGTTGCCATCATT | ACTCGTTGTACTTCCCATTGT |
| Bifidobacterium | TCGCGTCCGGTGTGAAAG | CCACATCCAGCGTCCAC |
| Akkermansia | CAGCACGTGAAGGTGGGGAC | CCTTGCGGTTGGCTTCAGAT |
| ropA-Ecoli | AGATGCCCTCGGTCTTTGT | GAGTAATTGATGAGCGTGCTG |

Table 4. List of primer sequences for RT-qPCR analysis of major tissues

| Name | Forward primer (5'-3') | Reverse primer (5'-3') |
| --- | --- | --- |
| GAPDH | AGGTCGGTGTGAACGGATTTG | TGTAGACCATGTAGTTGAGGTCA |
| COX-2 | CGAGTCGTTCTGCCAATA | CTGGTCGGTTTGATGCTA |
| NLRP3 | ATTACCCGCCCGAGAAAGG | TCGCAGCAAAGATCCACACAG |
| IL-1β | GGCTGGACTGTTTCTAATGC | ATGGTTTCTTGTGACCCTGA |
| IL-6 | GAAACCGCTATGAAGTTCCTCTCTG | TGTTGGGAGTGGTATCCTCTGTGA |
| iNOS | AGCCACCTTGGTGAAGGGA | TCCAAATCCAACGTTCTCCGT |
| TNF-α | GGGTGTTCATCCATTCTC | GGAAAGCCCATTTGAGT |
| ENaC-β | GGTCCTTATTGATGAGCG | TGAGAAGATGTTGGTGGC |
| AQP2 | TCATCGGTTCCCTCCTCTAC | GTTCCTCCCAGTCAGTGTCC |
| AQP3 | ACCCTGCCCGTGACTTTG | ACACCAGCGATGGAACCC |
| MUC2 | ATGCCCACCTCCTCAAAGAC | GTAGTTTCCGTTGGAACAGTGAA |
| MUC3 | CGTGGTCAACTGCGAGAATGG | CGGCTCTATCTCTACGCTCTCC |
| MMP-9 | GGACCCGAAGCGGACATTG | CGTCGTCGAAATGGGCATCT |
